# Transcriptome analysis of leaves, roots and flowers of *Panax notoginseng* identifies genes involved in ginsenoside and alkaloid biosynthesis

**DOI:** 10.1186/s12864-015-1477-5

**Published:** 2015-04-03

**Authors:** Ming-Hua Liu, Bin-Rui Yang, Wai-Fung Cheung, Kevin Yi Yang, He-Feng Zhou, Jamie Sui-Lam Kwok, Guo-Cheng Liu, Xiao-Feng Li, Silin Zhong, Simon Ming-Yuen Lee, Stephen Kwok-Wing Tsui

**Affiliations:** School of Biomedical Sciences, The Chinese University of Hong Kong, Hong Kong, China; Hong Kong Bioinformatics Centre, The Chinese University of Hong Kong, Hong Kong, China; State Key Laboratory of Quality Research in Chinese Medicine and Institute of Chinese Medical Sciences, Macao, China; Beijing Genomics Institute - Shenzhen, Shenzhen, 518083 China; School of Life Sciences, The Chinese University of Hong Kong, Hong Kong, China; Present correspondence: Groken Bioscience, Hong Kong, China

**Keywords:** *Panax notoginseng*, Transcriptome, Ginsenosides

## Abstract

**Background:**

*Panax notoginseng* (Burk.) F.H. Chen is one of the most highly valued medicinal plants in the world. The major bioactive molecules are triterpene saponins, which are also known as ginsenosides. However, its large genome size has hindered the assembly of a draft genome by whole genome sequencing. Hence, genomic and transcriptomic details about *P. notoginseng*, especially its biosynthetic pathways and gene expression in different parts of the plant, have remained largely unknown until now.

**Results:**

In this study, RNA sequencing of three different *P. notoginseng* tissues was performed using next generation DNA sequencing. After assembling the high quality sequencing reads into 107,340 unigenes, biochemical pathways were predicted and 9,908 unigenes were assigned to 135 KEGG pathways. Among them, 270 unigenes were identified to be involved in triterpene saponin biosynthesis. In addition, 350 and 342 unigenes were predicted to encode cytochrome P450s and glycosyltransferases, respectively, based on the annotation results, some of which encode enzymes responsible for the conversion of the triterpene saponin backbone into different ginsenosides. In particular, one unigene predominately expressed in the root was annotated as CYP716A53v2, which probably participates in the formation of protopanaxatriol from protopanaxadiol in *P. notoginseng*. The differential expression of this gene was further confirmed by real-time PCR.

**Conclusions:**

We have established a global transcriptome dataset for *P. notoginseng* and provided additional genetic information for further genome-wide research and analyses. Candidate genes involved in ginsenoside biosynthesis, including putative cytochrome P450s and glycosyltransferases were obtained. The transcriptomes in different plant tissues also provide invaluable resources for future study of the differences in physiological processes and secondary metabolites in different parts of *P. notoginseng*.

**Electronic supplementary material:**

The online version of this article (doi:10.1186/s12864-015-1477-5) contains supplementary material, which is available to authorized users.

## Background

*Panax notoginseng* (Burk.) F.H. Chen, which is popularly known as Sanqi or Tienchi Ginseng, is a species of the *Panax* genus in the *Araliaceae* family [1]. *P. notoginseng* has been cultivated for about 400 years in China. It was previously considered a variety of *Panax pseudo-ginseng*, but in 1948 it was defined as an independent species of the *Panax* genus by Chen Feng-Huai and is now officially named *Panax notoginseng* (Burk.) F.H. Chen. Nowadays, more than 85% of the *P. notoginseng* in the worldwide market is produced in the city of Wenshan, Yunnan Province, China.

*P. notoginseng* is present in several famous traditional Chinese medicinal products, such as *Yunnan Bai Yao* (a remedy for injury induced by trauma and bleeding) and *Pian Zai Huang* (a remedy for relieving pain and detoxification). It is also famous for its haemostatic properties [2]. The classification of *P. notoginseng* from the American Herbal Products Association is Class 2b and it is indicated in pregnancy because of possible haemostatic effects. It was reported that *P. notoginseng* extract administered to rats after cerebral ischaemia reduced infarct volume and inhibited inflammatory inhibitors such as inducible nitric oxide synthase and cyclooxygenase 2 via blocking of the NF-κB pathway [3], suggesting a neuroprotective effect. Moreover, saponins of *P. notoginseng* extract were able to modulate the expression of caspases and attenuate apoptosis in rats following focal cerebral ischaemia-reperfusion [4]. In KK-Ay diabetic mice injected with *P. notoginseng* extract, significantly lowered fasting blood glucose levels, improved glucose tolerance and lighter body weights were observed [5]. Besides the roots of *P. notoginseng*, total saponins extracted from caudexes and leaves have been commonly used for improving mental function, treating insomnia, and alleviating anxiety [6]. The flower buds of *P. notoginseng* are also used in clinics for treating hypertension, vertigo, tinnitus and acute faucitis in China [7].

Chemically, the main bioactive compounds found in *P. notoginseng* are saponins, which have diverse biological activities such as membrane-permeabilising, immunostimulating, hypocholesterolemic, anti-carcinogenic, and anti-microbial activities [8-10]. The *P. notoginseng*-derived triterpene saponins include 20(S)-protopanaxadiol and 20(S)-protopanaxatriol, which exhibit opposing wound healing and anti-tumour actions on the vascular system [11]. Notably, *P. notoginseng* also shares many similar chemical constituents with Asian ginseng (*P. ginseng* C.A. Mey) and American ginseng (*P. quinquefolius* L.) [12]. These *Panax* species have species-specific saponin constituents, e.g. pseudo-ginsenoside F11 is unique to American ginseng whereas ginsenoside Rg3 is only present in Asian ginseng [13]. More than 60 chemotypes of *P. notoginseng* classified according to the accumulation of different ginsenosides in roots, leaves and flowers have been reported [11,14]. *P. notoginseng* contains significantly higher amounts of ginsenosides Rg1 and Rb1 compared with other ginseng species, and the ratios of Rg1/Re and Rg1/Rb1 in *P. notoginseng* are the highest among ginseng species. In particular, notoginsenoside R1 has been identified in *P. notoginseng* but is absent in other ginseng species.

Several previous studies have suggested that the precursor molecules for triterpene saponin biosynthesis are isoprenoids, which are synthesized via the mevalonic acid (MVA) pathway, leading to the biosynthesis of 2,3-oxidosqualene [15]. This central molecule is then modified through various biochemical reactions of its triterpene skeleton, resulting in the production of various ginsenosides. Notably, ginsenosides can be isolated from different parts of *P. notoginseng*, e.g. the underground parts of *P. notoginseng* are rich in protopanaxatriol- and protopanaxadiol-type saponins, while leaves and flowers contain protopanaxadiol-type saponins only. On the other hand, the ginsenosides Rc, Rb2 and Rb3 are relatively abundant in aerial parts, compared with the underground parts of *P. notoginseng* [11,16]. Although considerable research has been done on the pharmacological activities of ginsenosides, to date very little is known about the ginsenoside biosynthetic pathway. Some candidate genes likely to be involved in hydroxylation or glycosylation of aglycones for triterpene saponin biosynthesis are cytochrome P450s (CYP450) and glycosyltransferases (GT), but no candidate has been identified for the cyclization step [17].

In addition, *P. notoginseng* is a shade plant and is commonly cultivated in mountain areas of Wenshan at altitudes of 1200–2000 m around 23.5°N, 104°E [18]. Because of the humid and warm environment, *P. notoginseng* is easily infected by pests and diseases, especially in the roots. Alkaloids, which have been identified in more than 4,000 plant species, play a role in protecting plants from pathogen and pest damage [19]. However, alkaloid-related genes in *P. notoginseng* have not been reported previously.

Transcriptomic and genomic data for *P. notoginseng* are very limited despite the pharmacological importance of this plant. Considering there are thousands of genes in its genome, only 435 mRNA sequences originating from *P. notoginseng* could be retrieved from the nucleotide databases of the National Centre for Biotechnology Information (NCBI). Over the last decade, next-generation DNA sequencing technology has provided a rapid and economical way to study the gene expression profiles of plant species. In this study, we established transcript databases for leaves, roots and flowers from 3-year-old *P. notoginseng*. Moreover, we identified genes encoding enzymes involved in triterpene saponin and alkaloid biosynthesis. Differentially expressed CYP450s and GTs in the three tissues are also reported.

## Results and discussion

### Sequencing and *de novo* assembly

To study the transcriptomes of *P. notoginseng*, leaves, roots and flowers were collected from 3-year-old plants. Total RNA was extracted from each part and then mRNA was isolated. Each sample was sequenced using the Illumina HiSeq™ 2000 platform. Sequencing yielded approximately 213 million 90-base pair (bp) paired end raw reads, or approximately 19 Gbp in total. We filtered out adapter sequences and reads that were shorter than 50 bp, and ultimately generated 5.8, 5.8 and 6.1 Gbp of high-quality (HQ) reads for leaves, roots and flowers, respectively. All of the HQ sequencing reads from the three organs were deposited in NCBI and can be accessed in the Sequence Read Archive (SRA) under the BioProject accession number PRJNA228978. The HQ reads of each sample library were assembled using the Trinity software [20] and the TGI Clustering Tool (TGICL) [21], followed by the Phrap assembler [22] to remove redundant Trinity-generated contigs. Finally, 128,665, 94,258 and 124,888 unigenes were obtained for leaves, roots and flowers, respectively. We also pooled the reads from all three organs together and repeated the above steps, resulting in 205,000 contigs and 107,340 unigenes with a mean length of 781 bp and 1,039 bp, respectively. The length distribution of contigs and unigenes is shown in Additional file 1. A summary of sequencing and assembly results is shown in Table 1.

**Table 1:** **Summary of Illumina sequencing and assembly of** ***P. notoginseng***

	**Leaves**	**Roots**	**Flowers**	**Three tissues together**
Number of HQ reads	65,041,040	65,258,974	68,125,310	198,425,324
Length of HQ reads (bp)	5,853,693,600	5,873,307,660	6,131,277,900	17,858,279,160
Number of contigs	167,954	140,265	184,389	205,000
Length of contigs (bp)	56,302,711	48,125,902	60,389,472	160,205,000
Average length of contigs (bp)	335	343	328	781
N50 of contigs (bp)	537	606	539	1,218
Number of unigene*	128,665	94,258	124,888	107,340
Length of unigene (bp)	76,176,975	60,684,037	92,995,924	111,620,756
Average length of unigene (bp)	592	644	745	1,039
N50 of unigene (bp)	912	1,025	1,232	1,526
Number of clusters	50,984	37,727	54,977	76,938
Number of singletons	77,681	56,531	69,911	30,402

*Total number of clusters and singletons.

Compared with a separate study of 4-year-old *P. notoginseng* [17], which generated 30,852 unigenes (14,005 contigs with mean length 581 bp and 16,847 singletons with mean length 343 bp), we present three times more and much longer unigenes from *P. notoginseng* roots (107,340 unigenes with mean length 1,039 bp). This is probably because of a 48-fold increase in sequencing throughput in this study. The large number of novel unigenes should cover the majority of genes in the *P. notoginseng* genome and provide a useful resource for future studies on this pharmacologically important plant.

### Annotation and differential expression of transcripts in different tissues

**Figure 1:**
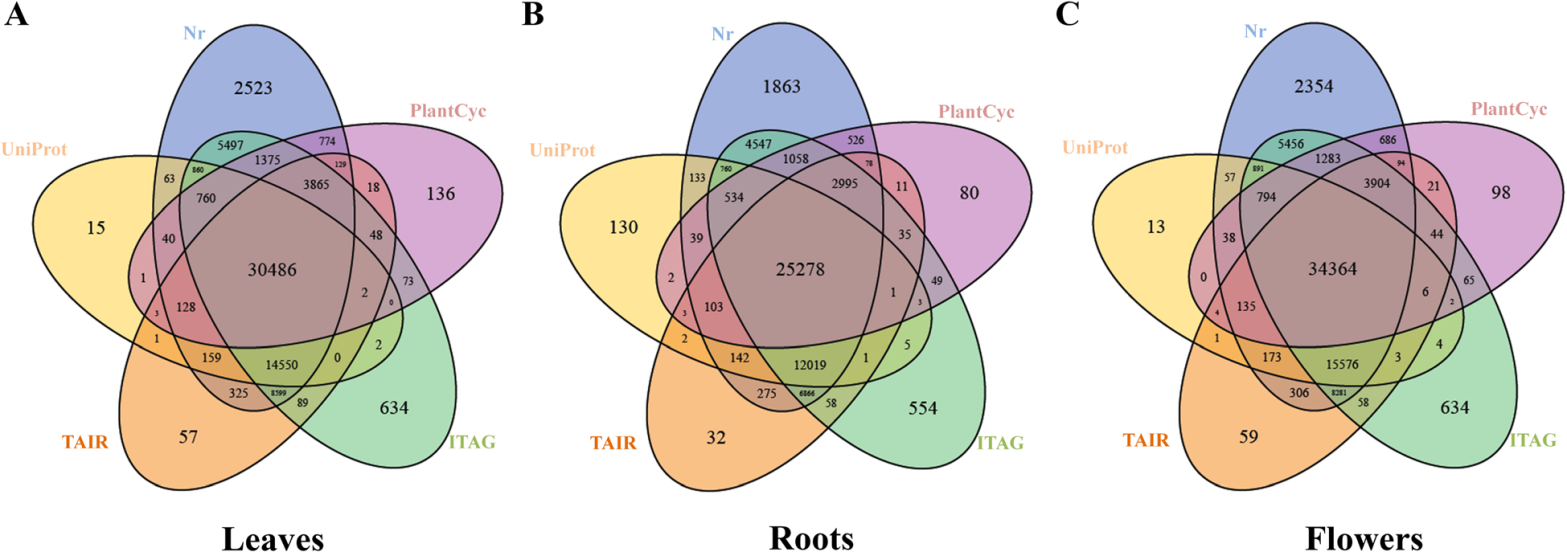
**Venn diagram of unigenes from leaves, roots and flowers annotated using various databases.** The Venn diagram shows the overlapping unigenes annotated in the Nr, Uniprot, TAIR, ITAG and PlantCyc databases in leaves **(A)**, roots **(B)** and flowers **(C)**.

**Figure 2:**
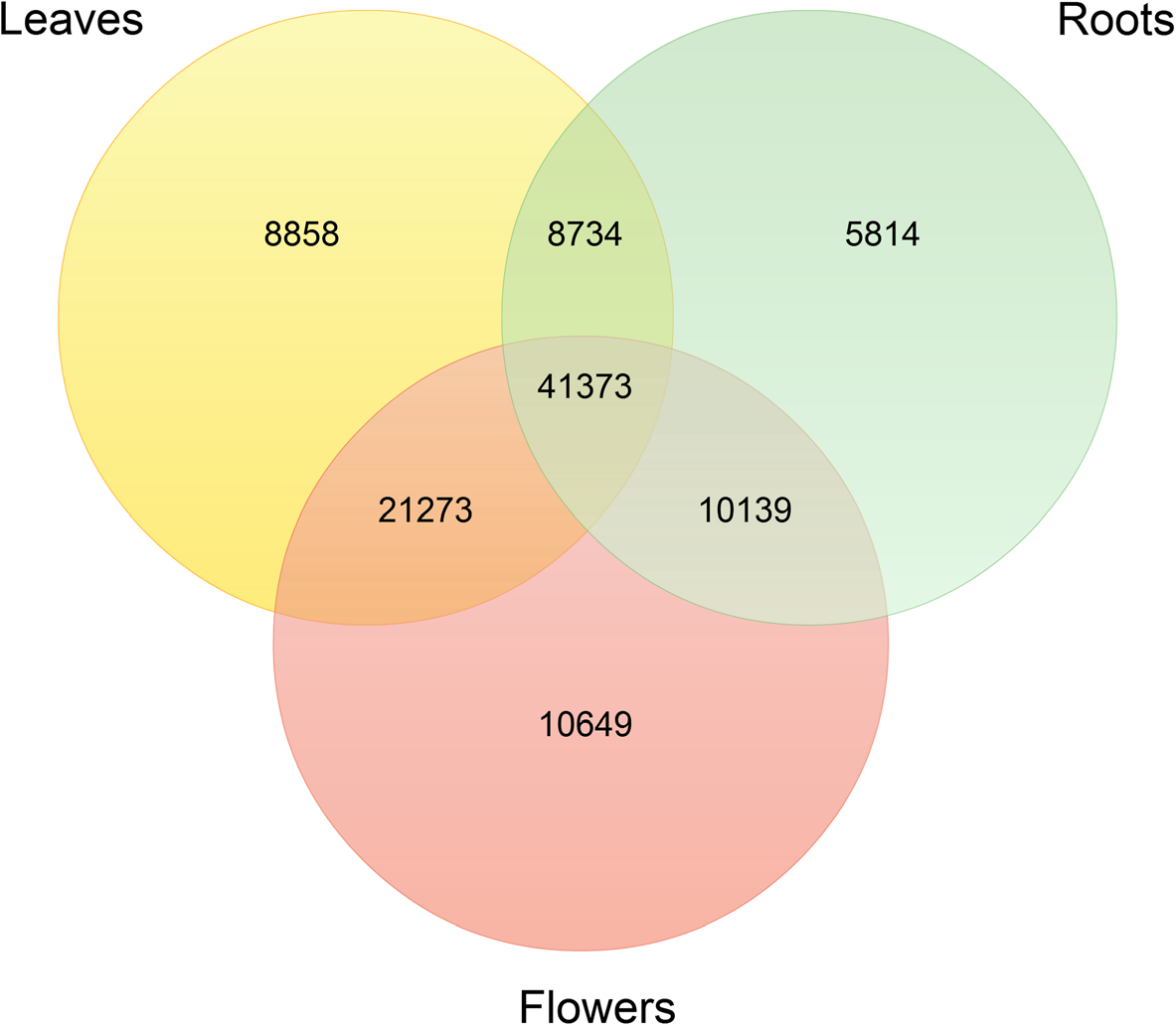
**Venn diagram of the unigenes in the leaves, roots and flowers of** ***P. notoginseng*** **.** The diagram shows the overlapping unigenes in the leaves, roots and flowers. A total 41,373 unigenes were found in all three tissues, while some unigenes only were found in specific tissues.

All unigenes from leaves, roots and flowers were annotated separately using BLAST searches against the NCBI non-redundant protein (Nr), UniProt protein, The Arabidopsis Information Resource (TAIR), tomato genome (ITAG) and PlantCyc public databases. Detailed counts of the annotated unigenes are presented in Additional file 2. In total, there were 71,212, 58,182 and 75,404 annotated unigenes for leaves, roots and flowers, respectively, with at least one significant match in the aforementioned public databases. Figure 1 shows the number of unigenes annotated by all databases. The annotation percentage for *P. notoginseng* unigenes (74,839 out of 107,340, 69.72%) was much higher than that for *P. ginseng* unigenes (94,535 out of 178,145, 53.06%) [23]. When the unigenes from different *P. notoginseng* tissues were compared, we found that 41,373 unigenes were shared by all three tissues (Figure 2). On the other hand, 8,858, 5,814 and 10,649 unigenes were specifically found in leaves, roots and flowers, respectively, with flowers having the highest number of unique unigenes.

We next investigated the transcriptomic similarities and differences between *P. notoginseng* and *P. ginseng* using BLAST searches of *P. notoginseng* unigenes against *P. ginseng* unigenes, with an E-value threshold of 1e-10 [23]. *P. ginseng* transcriptome contigs were obtained under accession number GAAG00000000 from the NCBI. The results showed that 77,470 unigenes of *P. notoginseng* had at least one match to the contigs of *P. ginseng*, suggesting that many of our unigenes are *P. notoginseng*-specific. Next, proteins encoded by our unigenes were predicted by the Trinity software and orthologous groups were identified using OrthoMCL [24], which groups orthologous proteins based on sequence similarity. A total of 64,742 protein sequences from 107,340 unigenes were clustered into 9,949 orthologous groups for *P. notoginseng*. For *P. ginseng*, a total of 29,289 protein sequences from 67,786 contigs were clustered into 8,959 orthologous groups. We found that 8,424 groups were common to both species, with 1,525 orthologous groups specific to *P. notoginseng*, and 535 groups specific to *P. ginseng*.

To evaluate the abundance of transcripts in specific organs, the high quality reads were mapped back to the transcriptome generated from all three plant tissues. Among the top 10 most expressed unigenes in the root transcriptome (Additional file 3), one of the most abundant genes was dammarenediol-II synthase (DS), which is an important player in triterpene saponin biosynthesis in *P. notoginseng* [25]. In addition, unigenes encoding cytochrome P450 CYP716A47, which is involved in the conversion of protopanaxadiol from dammarenediol-II, were also present at a very high level. This is consistent with a previous report about the co-expression of DS and CYP716A47 in *P. ginseng* [26]. Last but not least, reticuline oxidase-like protein-like isoform 1 is a predicted protein with sequence similarity to reticuline oxidase, which is involved in forming benzophenanthridine alkaloids as a pathogenic attack response.

### Identification of genes involved in triterpene saponin biosynthesis

The Kyoto Encyclopaedia of Genes and Genomes (KEGG) is a tool for functional classification and pathway assignment based on gene-associated biochemical pathways. In total, 9,908 unigenes from all three tissues having enzyme commission numbers were assigned to 135 KEGG pathways (Additional file 4). The cluster for metabolism represented the largest group, with most unigenes involved in carbohydrate metabolism, amino acid metabolism, nucleotide metabolism and metabolism of cofactors and vitamins. Among them, 616 unigenes were involved in the biosynthesis of various secondary metabolites (Table 2), with unigenes involved in phenylpropanoid biosynthesis forming the largest group, followed by terpenoid backbone biosynthesis.

**Table 2:** **Number of unigenes related to secondary metabolites in** ***P. notoginseng***

**Secondary metabolites biosynthesis pathways**	**Pathway ID**	**Leaves**	**Roots**	**Flowers**
Anthocyanin biosynthesis	ko00942	10	7	14
Caffeine metabolism	ko00232	12	12	20
Carotenoid biosynthesis	ko00906	15	5	32
Cutin, suberin and wax biosynthesis	ko00073	14	12	15
Diterpenoid biosynthesis	ko00904	8	3	12
Flavone and flavonol biosynthesis	ko00944	3	2	5
Flavonoid biosynthesis	ko00941	23	21	33
Indole alkaloid biosynthesis	ko00901	8	13	11
Isoquinoline alkaloid biosynthesis	ko00950	57	32	51
Limonine and pinene degradation	ko00903	13	10	15
Monoterpenoid biosynthesis	ko00902	4	6	6
Nicotinate and nicotinamide metabolism	ko00760	37	39	48
Phenylpropanoid metabolism	ko00940	88	100	154
Sesquiterpenoid and triterpenoid biosynthesis	ko00909	19	15	13
Steroid biosynthesis	ko00100	27	18	42
Stibenoid diarylhepatanoid and gingerol biosynthesis	ko00945	2	1	2
Terpenoid backbone biosynthesis	ko00900	91	56	56
Tropane, piperidine and pyridine alkaloid biosynthesis	ko00960	57	47	66
Ubiquinone and other terpenoid-quinone biosynthesis	ko00130	45	20	31
Zeatin biosynthesis	ko00908	10	6	14

**Figure 3:**
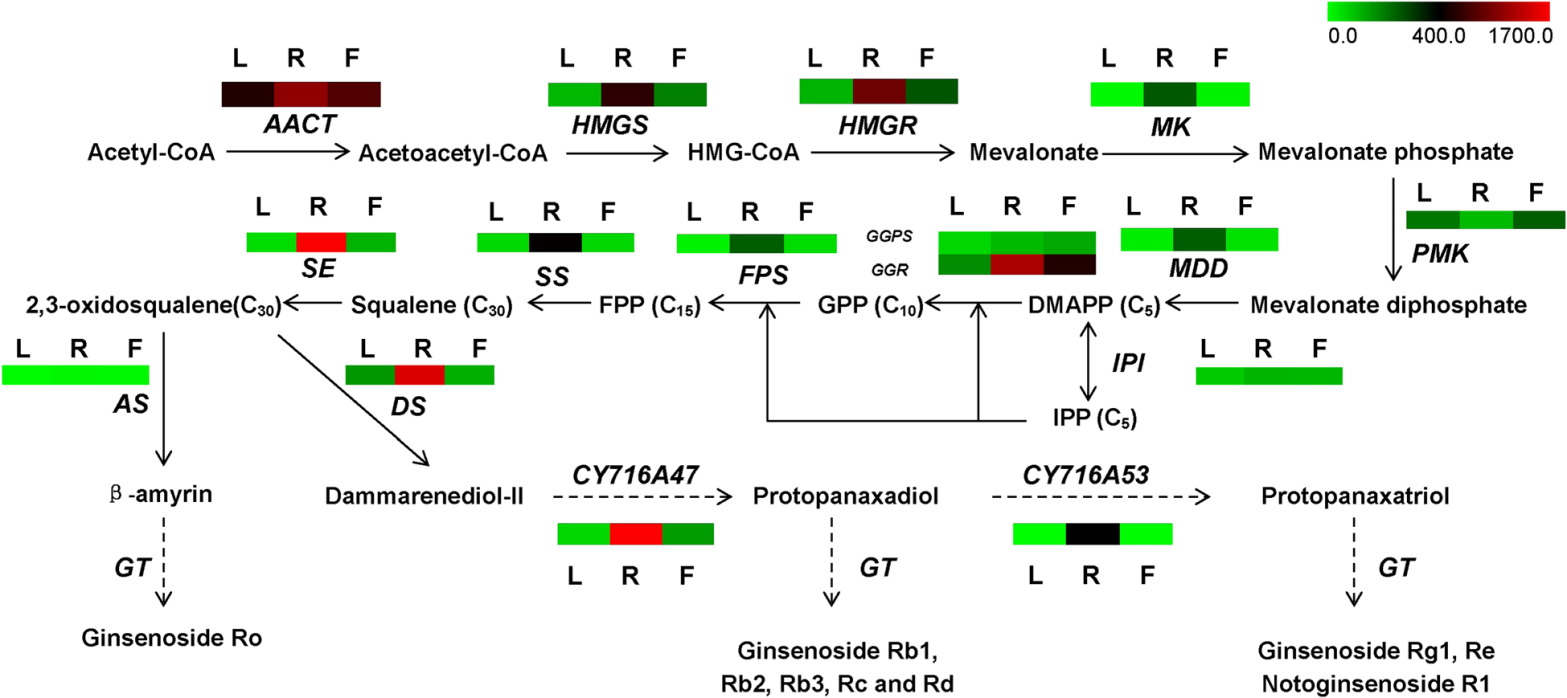
**Putative ginsenoside biosynthesis in** ***P. notoginseng*** **.** Enzymes found in this study are shown between the reactions. The expression of genes encoding these enzymes in the leaves (L), roots (R) and flowers (F) is shown by heatmap. The genes were mapped using RPKM values, colour coded by increasing relative expression. The broken arrow represents a putative ginsenoside biosynthesis step involving CYP450s and GTs. Abbreviations: AACT, acetyl-CoA acetyltransferase; HMGS, HMG-CoA synthase; HMGR, HMG-CoA reductase; MK, mevalonate kinase; PMK, phosphomevalonate kinase; MDD, mevalonate diphosphate decarboxylase; IPI, isopentenylpyrophosphate isomerase; GGPS, geranylgeranyl pyrophosphate synthase; GGR, geranylgeranyl diphosphate synthase; FPS, farnesyl diphosphate synthase; SS, squalene synthase; SE, squalene epoxidase; AS, β-amyrin synthase; DS, dammarenediol-II synthase; CYP450, cytochrome P450; GT, glycosyltransferase.

**Figure 4:**
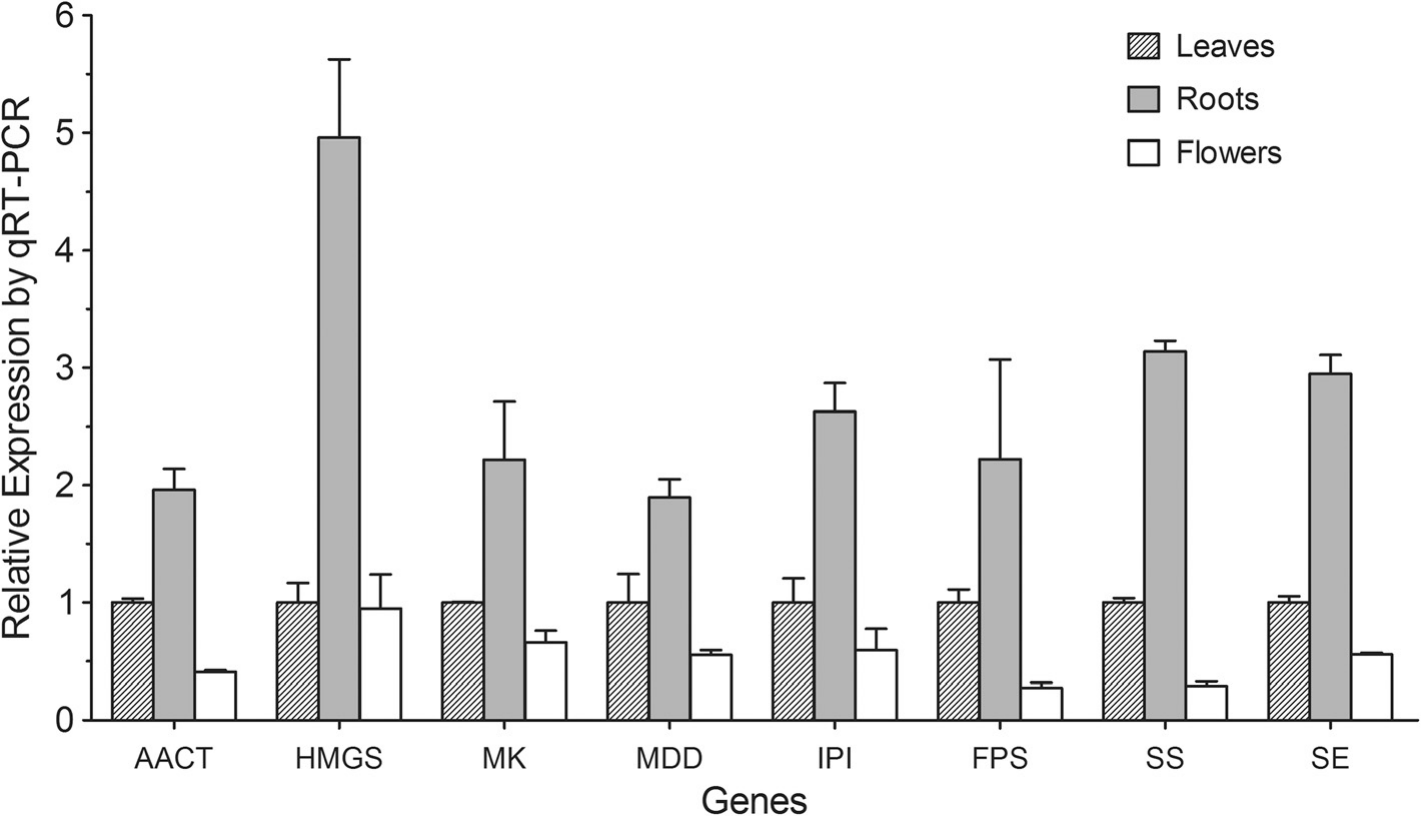
**Real-time PCR analysis of selected genes involved in triterpene saponin biosynthesis.** Real-time PCR was used to validate the expression levels of selected genes revealed by RNA-seq. Abbreviations: AACT, acetyl-CoA acetyltransferase; HMGS, HMG-CoA synthase; MK, mevalonate kinase; MDD, mevalonate diphosphate decarboxylase; IPI, isopentenylpyrophosphate isomerase; FPS, farnesyl diphosphate synthase; SS, squalene synthase; SE, squalene epoxidase.

Triterpene saponins are synthesized by terpenoid backbone biosynthesis, followed by sesquiterpenoid and triterpenoid biosynthesis. According to the putative pathway, specific CYP450s and GTs are involved in the formation of various ginsenosides (Figure 3). From our annotation results, 270 unigenes were identified to encode all of the known enzymes involved in triterpene saponin biosynthesis. There were multiple unigenes annotated to the same enzyme, which may represent different members of the same gene family. Table 3 shows the reads per kilobase of transcript per million reads mapped (RPKM) values of genes encoding enzymes involved in triterpene saponin biosynthesis in leaves, roots and flowers. The RPKMs of all annotated isoforms for the same gene were summed as the RPKM of that gene. We identified 12 out of 14 full-length cDNA encoding enzymes involved in triterpene saponin biosynthesis and their corresponding accession numbers in NCBI nucleotide databases are listed in Table 4. A heat map of differentially expressed genes involved in triterpene saponin biosynthesis is shown in Figure 3. Significant differential expression of these genes could be recognized in different *P. notoginseng* tissues, and most of the genes involved in triterpene saponin biosynthesis showed higher expression in roots compared with leaves or flowers. The expression levels of selected genes (8 out of 14) in each part were validated by real-time PCR (Figure 4).

**Table 3:** **Discovery of unigenes involved in triterpene saponin biosynthesis in** ***P. notoginseng***

**Enzymes name**	**EC number**	**Abbreviation**	**Leaves**	**Roots**	**Flowers**
Acetyl-CoA acetyltransferase	2.3.1.9	AACT	587.76	1142.49	816.36
Hydroxymethyl glutaryl CoA synthase	2.3.3.10	HMGS	110.12	634.67	200.10
3-hydroxy-3-methylglutaryl-coenzymeA reductase	1.1.1.34	HMGR	117.75	961.07	263.98
Mevalonate kinase	2.7.1.36	MK	9.42	261.79	19.36
Phosphomevalonate kinase	2.7.4.2	PMK	211.09	107.73	251.65
Mevalonate diphosphosphate decarboxylase	4.1.1.33	MDD	27.24	252.63	46.67
Isopentenylpyrophosphate isomerase	5.3.3.2	IPI	81.66	115.06	112.65
Geranylgeranyl pyrophosphate synthase	2.5.1.29	GGPS	63.48	106.71	136.29
Geranylgeranyl diphosphate synthase	2.5.1.1	GGR	178.22	1283.19	586.18
Farnesyl diphosphate synthase	2.5.1.10	FPS	18.93	252.86	54.37
Squalene synthase	2.5.1.21	SS	57.08	435.07	63.94
Squalene epoxidase	1.14.13.132	SE	53.97	1690.83	118.00
β-amyrin synthase	5.4.99.39	AS	6.47	12.30	8.34
Dammarenediol-II synthase	4.2.1.125	DS	162.68	1526.21	124.10

The values in different organs indicate the reads per kilobase of transcript per million reads mapped (RPKM).

**Table 4:** **Genes involved in triterpene saponin biosynthesis in** ***P. notoginseng***

**Gene name**	**Abbreviation**	**Length of mRNA (bp)**	**Location of CDS**	**Accession number**
Acetyl-CoA C-acetyltransferase	AACT	1816	194-1420	KJ804173
3-hydroxy-3-methylglutaryl coenzyme A synthase	HMGS	1986	388-1797	KJ804167
3-hydroxy-3-methylglutaryl coenzyme A reductase	HMGR	3947	198-1967	KJ804166
Mevalonate kinase	MK	1767	207-1370	KJ804176
Phosphomevalonate kinase	PMK	2078	277-1806	KJ804170
Mevalonate diphosphate decarboxylase	MDD	2658	386-1648	KJ804169
Isopentenyl diphosphate isomerase	IPI	1246	119-1042	KJ804168
Geranylgeranyl diphosphate synthase	GGR	1826	368-1474	KJ804178
Geranylgeranyl pyrophosphate synthase*	GGPS	1023	1-885	KJ804179
Farnesyl diphosphate synthase	FPS	1389	141-1169	KJ804175
Squalene synthase	SS	1719	284-1531	KJ804172
Squalene epoxidase	SE	2054	104-1726	KJ804171
Beta-amyrin synthase*	AS	2291	1-2130	KJ804177
Dammarenediol-II synthase	DS	2631	117-2426	KJ804174

*Partial CDS at the 5′ end.

Luo *et al*. [17] previously analysed the root transcriptome of 4-year-old *P. notoginseng* and discovered many partial cDNAs encoding enzymes involved in triterpene saponin biosynthesis. However, hydroxymethyl glutaryl CoA synthase (HMGS), a key enzyme of triterpene saponin biosynthesis, was absent in their transcriptome. In contrast, the HMGS gene was abundant in our transcriptomes, probably because of the much greater sequencing depth achieved in our study. Therefore, compared with other published transcriptomes, the transcriptomes reported in this study will be more useful for cloning important genes involved in secondary metabolite biosynthetic pathways of *P. notoginseng*. Moreover, β-amyrin synthase (AS), a key enzyme for oleanane-type ginsenoside biosynthesis, was also found in this study. Notably, AS was discovered in all three tissues of 3-year-old *P. notoginseng* (Table 3).

### Identification of genes related to biosynthesis of different ginsenosides

There are published reports showing that different parts of *P. notoginseng* differ in the synthesis of triterpene saponins. Roots are rich in protopanaxatriol- and protopanaxadiol-type saponins whereas leaves and flowers contain protopanaxadiol-type saponins only [11]. It has been suggested that putative candidate genes involved in triterpene saponins biosynthesis are mainly CYP450s and GTs, which may account for the synthesis and accumulation of triterpene saponins in specific organs [27,28]. In this study, 350 and 342 members of the CYP450 and GT gene families, respectively, were identified (Additional files 5 and 6).

**Figure 5:**
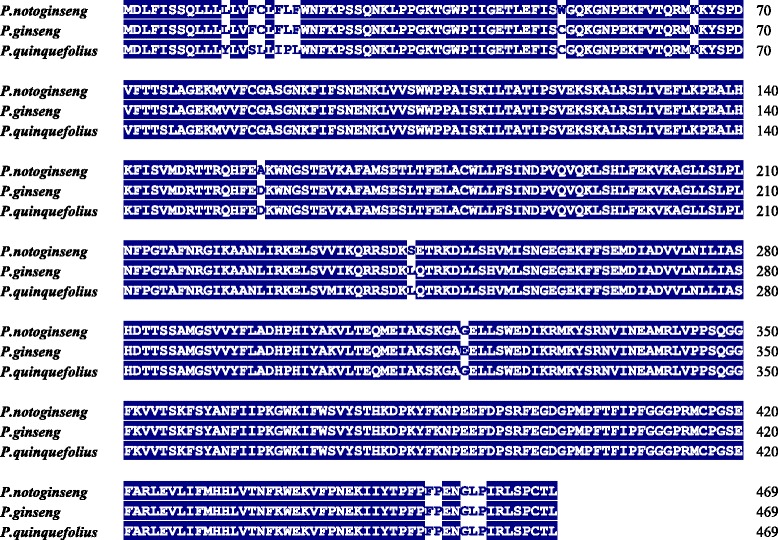
**Alignment of CYP716A53v2 amino acid sequences from** ***P. notoginseng*** **,** ***P. ginseng*** **and** ***P. quinquefolium*** **.** Alignment results showed 98% identity between *P. notoginseng* and *P. ginseng*, 96% identity between *P. notoginseng* and *P. quinquefolius*, and 97% identity between *P. ginseng* and *P. quinquefolius.*

**Figure 6:**
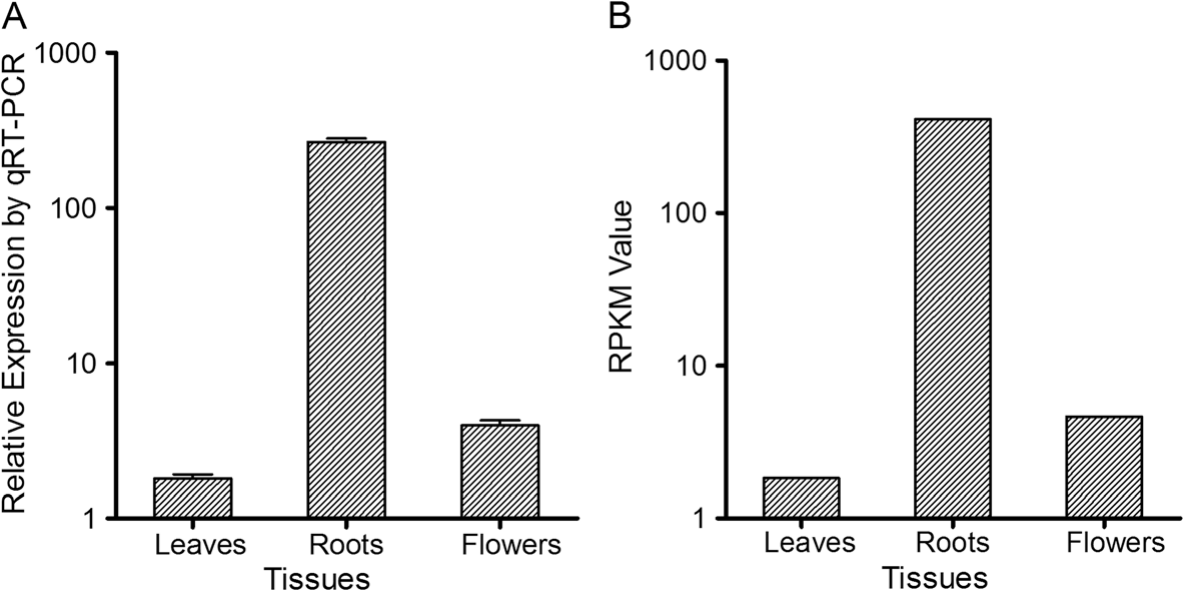
**Expression levels of target CYP450s in different** ***P. notoginseng*** **tissues.** Comparison of relative abundance of target CYP450 unigenes in the leaf, root and flower according to real time PCR **(A)** and RPKM values **(B)**.

It has been reported that the cytochrome P450 CYP716A53v2 participates in the formation of protopanaxatriol from protopanaxadiol in *P. ginseng* [29]. In our transcriptome, one unigene annotated as CYP716A53v2 was found and subsequent sequence analysis showed that it was the full-length homologue in *P. notoginseng*. Figure 5 shows the alignment of the predicted protein sequences of CYP716A53v2 from *P. notoginseng*, *P. ginseng* and *P. quinquefolius*. The accession numbers of the sequences are JX036031 for *P. ginseng* and KC190491 for *P. quinquefolius* in the GenBank database. All three CYP450 genes had 469 amino acid residues and showed 98% identity between *P. notoginseng* and *P. ginseng*, 96% identity between *P. notoginseng* and *P. quinquefolius*, and 97% identity between *P. ginseng* and *P. quinquefolius*. It was also very encouraging to find that the expression level of the putative CYP716A53v2 in the root (RPKM value 414.91) was much higher than that in the leaf (RPKM value 1.83, *P* < 0.001) or flower (RPKM value 4.62, *P* < 0.001) of *P. notoginseng* (Figure 6). To confirm the differential expression of the putative CYP716A53v2 gene, we analysed its expression level in different tissues by real-time PCR. The result obtained was consistent with the RPKM values, showing that the expression of the putative CYP716A53v2 in roots was significantly higher than in leaves or flowers (Figure 6). This may explain, at least partially, why the root has a much higher concentration of protopanaxatriol-type saponins. Moreover, this result also indicates that the abundance of target genes in our transcriptomes closely reflects the actual gene expression level.

### Identification of candidate genes involved in alkaloid biosynthesis

Alkaloids, which are found in about 20% of plant species, are a diverse group of low-molecular-weight compounds acting as poisonous agents in the defence of plants against herbivores and pathogens [19]. *P. notoginseng* grows sub-optimally in direct sunlight and so is often planted under tree canopies, but the shady and humid growing conditions favour infection by numerous phytopathogens, which can cause root rot, black spot or round spot diseases [18].

**Figure 7:**
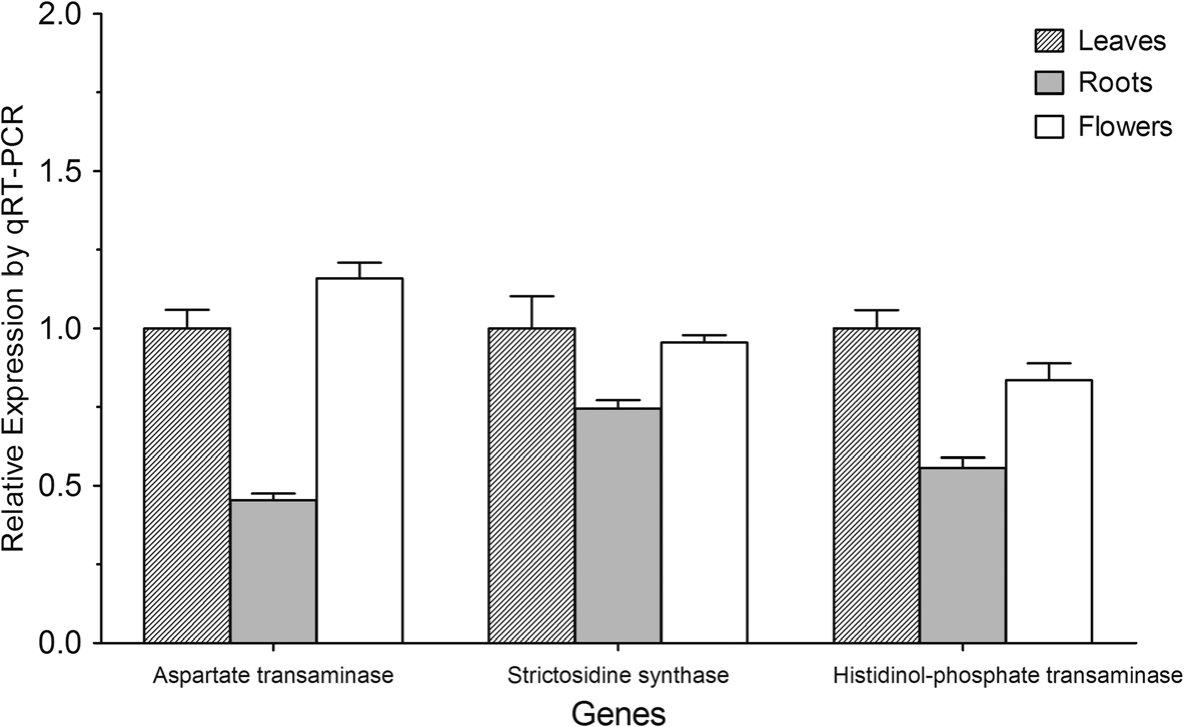
**Real-time PCR analysis of genes involved in the alkaloid pathway.** The expression levels of selected genes in the alkaloid pathway were validated by real-time PCR.

In this study, three pathways involved in alkaloid biosynthesis were found in our transcriptomes (Table 2), including isoquinoline alkaloid biosynthesis (KEGG pathway entry ko00950), indole alkaloid biosynthesis (ko00901) and tropane, piperidine and pyridine alkaloid biosynthesis (ko00945). The annotation results from the three transcriptomes were used to identify genes encoding enzymes involved in various alkaloid biosynthetic pathways. In total, 72 unigenes were assigned to six enzymes involved in alkaloid biosynthesis (Additional file 7). According to the RPKM values of the genes encoding enzymes involved in alkaloid biosynthesis in leaves, roots and flowers shown in Table 5, most of the enzymes were expressed at the lowest level in roots, in particular polyphenol oxidase (PPO), which has a role in plant resistance to stress and pathogens. It is notable that wounding and herbivore attacks have also been shown to induce PPO activity [30]. PPO activation is thought to involve proteolytic processing, but many mature PPOs appear to remain in a latent form [31]. Root rot disease is the most damaging disease that can plague *P. notoginseng* over its long growth period, leading to production loss and quality reduction. It is mainly caused by fungal pathogens such as *Cylindrocarpon destructans*, *Cylindrocarpon didymum* and *Fusarium solani* [32]. In this study, the expression levels of aspartate transaminase, strictosidine synthase and histidinol-phosphate transaminase in different tissues were validated by real-time PCR (Figure 7). Further investigation of the relationship between alkaloid biosynthesis genes and plant defence should provide novel insights into the complex disease resistance mechanisms of *P. notoginseng*.

**Table 5:** **Discovery of unigenes involved in alkaloid biosynthesis in** ***P. notoginseng***

**Enzymes**	**EC number**	**Leaves**	**Roots**	**Flowers**
Aspartate transaminase	2.6.1.1	234.19	173.03	256.16
Primary-amine oxidase	1.4.3.21	153.62	125.11	211.14
Strictosidine synthase	4.3.3.2	140.37	35.36	121.67
Polyneuridine-aldehyde esterase	3.1.1.78	21.37	21.74	34.13
Histidinol-phosphate transaminase	2.6.1.9	26.40	20.85	26.16
Polyphenol oxidase	1.10.3.1	143.53	17.70	145.64

The values in different organs indicate the reads per kilobase of transcript per million reads mapped (RPKM).

## Conclusions

*P. notoginseng* is a widely used medicinal herb. In this study, leaf, root and flower transcriptomes from *P. notoginseng* are presented. The resulting unigene dataset will provide a large number of transcripts for gene discovery and genetic analyses in this medicinal plant. Notably, many genes involved in triterpene saponin biosynthesis were identified in this study. More importantly, a large number of unigenes were annotated as CYP450s and GTs. In summary, this study provides comprehensive information on the transcriptional regulation of functionally important genes in *P. notoginseng*.

## Methods

### Plant materials

Growing 3-year-old *P. notoginseng* was randomly collected from the field of a *P. notoginseng* commercial planting base in Wenshan County, Yunnan Province, China on September 2, 2012. The highest/lowest temperature on that day was 28/20°C, and the relative humidity was 78%. The soil of the field was a sandy loam soil with a pH value of 5.5–7.0. After cleansing, the leaves, roots and flowers were collected separately, cut into small pieces, immediately frozen in liquid nitrogen, and stored at −80°C until further processing.

### RNA extraction

mRNA isolation, cDNA library construction and sequencing were performed by the Beijing Genomics Institute (BGI) (Shenzhen, China). Briefly, total RNA was extracted from each tissue using TRIzol reagent (Invitrogen, Burlington, ON, Canada) and digested with DNase I (Takara, Dalian, China) according to the manufacturer’s protocol. Next, Oligo (dT) magnetic beads were used to isolate mRNA from the total RNA. By mixing with fragmentation buffer, the mRNA was then broken into short fragments.

### cDNA synthesis and sequencing

The cDNA was synthesized using the mRNA fragments as templates. The short fragments were purified and resolved with EB buffer for end repair and single nucleotide A (adenine) addition, and then connected with adapters. Suitable fragments were selected for PCR amplification as templates. During the quality control steps, an Agilent 2100 Bioanalyzer (Agilent Technologies, Redwood City, CA, USA) and ABI StepOnePlus Real-Time PCR System (Life Technologies, Grand Island, NY, USA) were used for quantification and qualification of the sample library. Each cDNA library was sequenced in a single lane of the Illumina HiSeq^TM^ 2000 system using paired end protocols according to the manufacturer’s instructions at the Beijing Genomics Institute (BGI) (Shenzhen, China). The amount of reads generated per sample was 5–8 Gb to obtain deep coverage of transcripts for *de novo* assembly [33]. As determined by SAMtools [34], the average depth of the generated *P. notoginseng* transcriptomes was over 130 × .

### *De novo* assembly and sequence annotation

The raw reads dataset was first processed to remove the reads with adaptors or containing more than five unknown (‘N’) nucleotides. Next, the low quality reads (defined as reads having more than 20% of bases with quality ≤ 10) were trimmed. We used the Trinity software (release-20121005) [20] for *de novo* assembly of the high-quality cleaned reads, and then used TGICL [21] followed by the Phrap [22] assembler to remove the redundant Trinity-generated contigs.

The resulting unigenes were annotated by BLASTx [35] searches against the NCBI nr (ftp://ftp.ncbi.nih.gov/blast/db/FASTA/nr.gz), UniProt protein (http://www.uniprot.org/downloads), TAIR (http://www.arabidopsis.org/index.jsp), ITAG (http://solgenomics.net/organism/Solanum_lycopersicum/genome) and PlantCyc (ftp://ftp.plantcyc.org/Pathways/BLAST_sets/) databases with a cut-off value of 1e-5. The top hit was extracted for each unigene.

Groups of orthologous proteins were identified using the OrthoMCL algorithm [24]. To gain an overview of gene networks, KEGG pathway information was assigned to each unigene, based on similarity with the KEGG database [36] using a BLAST search with a cut-off value of 1e-5. The KEGG analysis output included enzyme commission (EC) numbers and KEGG orthology (KO) numbers.

### Digital gene expression profiling

The high-quality reads were aligned to the assembled unigenes with the BWA program [37]. An RPKM value was calculated for each unigene in each tissue of *P. notoginseng*. The RPKMs of all annotated isoforms for the same gene were summed as the RPKM of that gene. Differential expression of unigenes was calculated with a threshold of *P* value < 0.001 and 2-fold change.

### Real-time PCR analysis

RNA was isolated from different *P. notoginseng* tissues and reverse-transcribed to single-strand cDNA using the Super Script™ III First-Strand Synthesis System (Invitrogen™, USA). Quantitative reactions were performed on the Real-Time PCR Detection System (ABI 7500, Applied Biosystems, USA) using SYBRR Premix Ex Taq™ II (Takara Biotechnology, China). The reaction mixture (20 μL) contained 2× SYBRR Premix Ex Taq™ II, 0.4 μM each of the forward and reverse primers, and 2 μL of template cDNA. PCR amplification was performed under the following conditions: 95°C for 30 s, followed by 40 cycles of 95°C for 5 s and 60°C for 34 s, and with a dissociation stage of 95°C for 15 s, 60°C for 60 s and 95°C for 15 s. All primers used in this study are listed in Additional file 8. The relative gene expression was calculated with the ^ΔΔ^CT method. For each sample, the mRNA levels of the target genes were normalized to that of the actin mRNA. These experiments were repeated using three biological replications.

### Ethical statement

There is no conflict of interest. An exemption from requiring ethics approval has been granted from the Joint Chinese University of Hong Kong - New Territories East Cluster Clinical Research Ethics Committee.

### Availability of supporting data

All RNA sequences and raw reads data are available under accession numbers KJ804166 to KJ804179, KF935232 and BioProject accession PRJNA228978.

## Additional files

Additional file 1:
**Length distribution of contigs and unigenes from leaves, roots and flowers of** ***P. notoginseng.*** PDF document of the length distribution of contigs and unigenes. Additional file 2:
**Summary of annotation statistics of the leaves, roots and flowers using various databases.** PDF document of the summary of annotation. Additional file 3:
**Top 10 most highly expressed transcripts in the** ***P. notoginseng*** **root transcriptome.** PDF document of the annotation results of the top 10 highly expressed unigenes in the root. Additional file 4:
**Number of unigenes assigned to KEGG biochemical pathways in** ***P. notoginseng.*** PDF document of the number of unigenes assigned to KEGG biochemical pathways. Additional file 5:
**Summary of unigenes annotated as CYP450.** XLSX document of the summary of unigenes annotated as CYP450. Additional file 6:
**Summary of unigenes annotated as glycosyltransferase.** XLSX document of the summary of unigenes annotated as glycosyltransferase. Additional file 7:
**Summary of unigenes encoding enzymes involved in the alkaloid pathway.** XLSX document of the annotation results of unigenes encoding enzymes involved in the alkaloid pathway. Additional file 8:
**List of real-time PCR primer sequences.** PDF document of the list of real-time PCR primer sequences.

## Abbreviations

AS: β-amyrin synthase
BLAST: Basic local alignment search tool
bp: Base pair
BWA: Burrows-Wheeler Alignment Tool
cDNA: Complementary DNA
CYP450: Cytochrome P450
DS: Dammarenediol-II synthase
EC: Enzyme commission
GT: Glucosyltransferase
HMGS: Hydroxymethyl glutaryl CoA synthase
ITAG: Tomato genome database
KEGG: Kyoto encyclopaedia of genes and genomes
KO: KEGG orthology
MVA: Mevalonic acid
NCBI: National Centre for Biotechnology Information
Nr: Non-redundant protein database
PPO: Polyphenol oxidase
RPKM: Reads per kilobase of transcript per million reads mapped
SRA: Sequence Read Archive
TAIR: The Arabidopsis Information Resource
TGICL: TGI Clustering Tool
