# Supplementary material for: Transcriptome analysis of leaves, roots and flowers of Panax notoginseng identifies genes involved in ginsenoside and alkaloid biosynthesis
Source: BMC Genomics. 2015 Apr 3;16(1):265. doi: 10.1186/s12864-015-1477-5 (PMC4399409; doi:10.1186/s12864-015-1477-5)
Supplement: Additional file 1: — Length distribution of contigs and unigenes from leaves, roots and flowers of P. notoginseng. PDF document of the length distribution of contigs and unigenes. [file 12864_2015_1477_MOESM1_ESM.pdf]

A

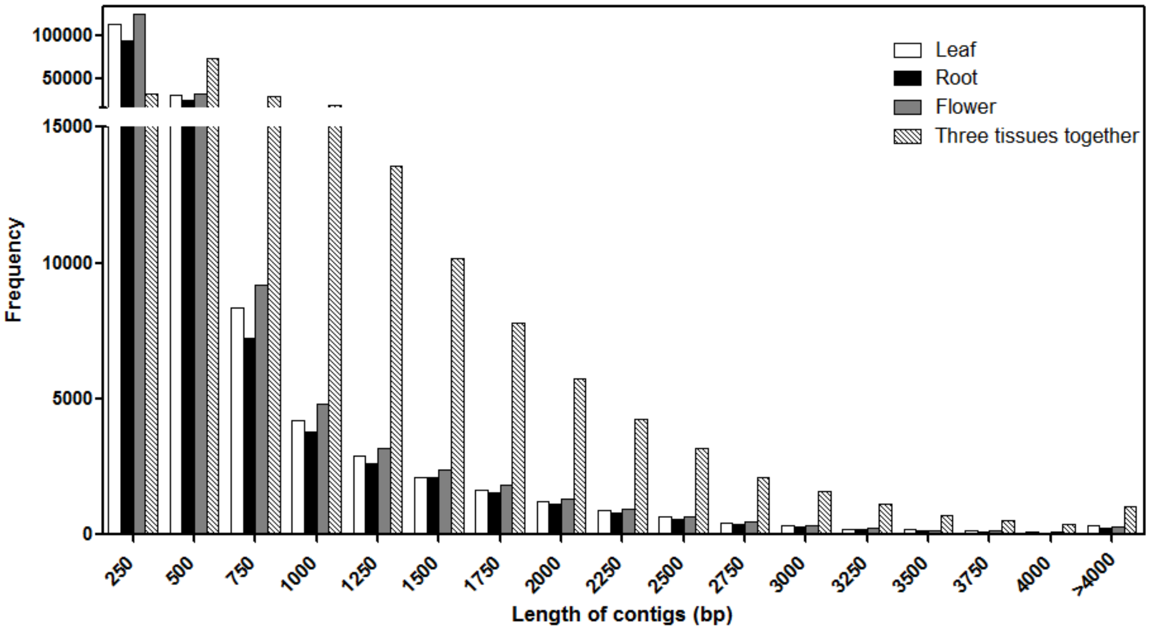

B

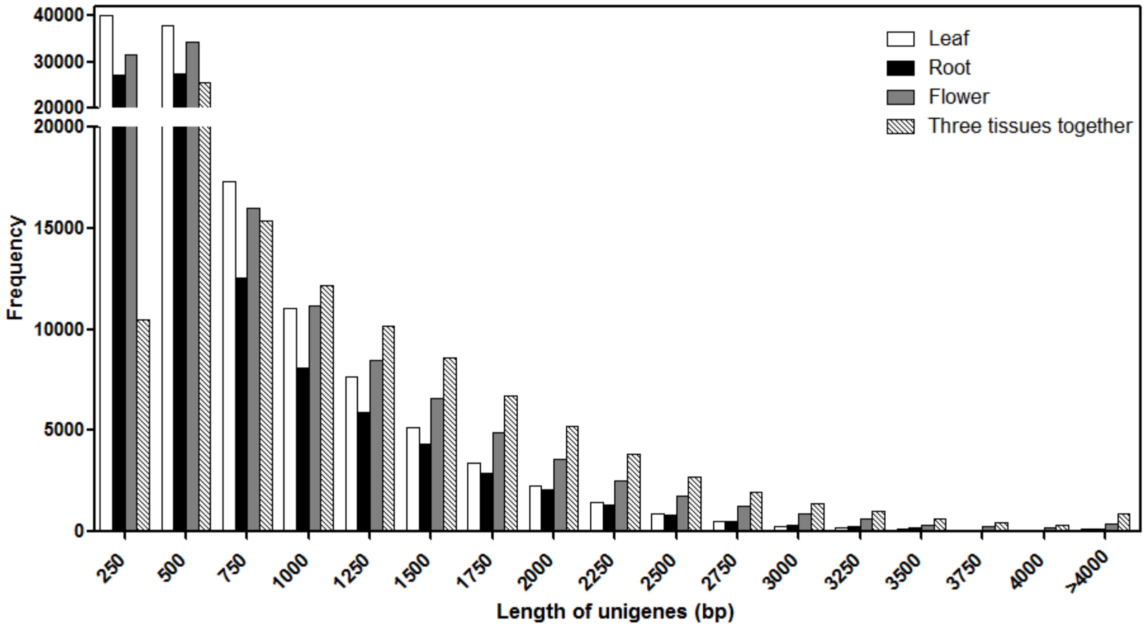

Additional file 1 - Length distribution of contigs and unigenes from leaves, roots and flowers of *P. notoginseng*.

A. Histogram of contigs lengths in three tissues. B. Histogram of unigenes length in three tissues.
