# Supplementary material for: Transcriptome analysis of leaves, roots and flowers of Panax notoginseng identifies genes involved in ginsenoside and alkaloid biosynthesis
Source: BMC Genomics. 2015 Apr 3;16(1):265. doi: 10.1186/s12864-015-1477-5 (PMC4399409; doi:10.1186/s12864-015-1477-5)
Supplement: Additional file 2: — Summary of annotation statistics of the leaves, roots and flowers using various databases. PDF document of the summary of annotation. [file 12864_2015_1477_MOESM2_ESM.pdf]

Additional file 2 - Summary of annotation statistics of the leaves, roots and flowers using various databases

	Leaves		Roots		Flowers		Three tissues together	
	Number of sequences	Annotation percentage (%)	Number of sequences	Annotation percentage (%)	Number of sequences	Annotation percentage (%)	Number of sequences	Annotation percentage (%)
Nr	70,133	54.51	57,216	60.70	74,392	59.57	74,115	69.05
UniProt	47,070	36.58	39,155	41.54	52,061	41.69	54,632	50.90
TAIR	58,459	45.44	47,899	50.82	63,029	50.47	64,248	59.85
ITAG	66,840	51.95	54,763	58.10	71,365	57.14	71,747	66.84
PlantCyc	37,838	29.41	30,796	32.67	41,538	33.26	43,713	40.72
Total annotation	71,212	55.35	58,182	61.73	75,404	60.38	75,020	69.89
Total unigenes	128,665		94,258		124,888		107,340	
