# Supplementary material for: Transcriptome analysis of leaves, roots and flowers of Panax notoginseng identifies genes involved in ginsenoside and alkaloid biosynthesis
Source: BMC Genomics. 2015 Apr 3;16(1):265. doi: 10.1186/s12864-015-1477-5 (PMC4399409; doi:10.1186/s12864-015-1477-5)
Supplement: Additional file 3: — Top 10 most highly expressed transcripts in the P. notoginseng root transcriptome. PDF document of the annotation results of the top 10 highly expressed unigenes in the root. [file 12864_2015_1477_MOESM3_ESM.pdf]

Additional file 3 - Top 10 most highly expressed transcripts in the *P. notoginseng* root transcriptome

Unigene name	No. of reads	Annotation
CL41816Contig1	4459833	RNase-like major storage protein [*Panax ginseng*]
CL29687Contig1	3681462	RNase-like major storage protein [*Panax ginseng*]
CL9334Contig1	900906	PREDICTED: reticuline oxidase-like protein-like isoform 1 [*Glycine max*]
CL33832Contig1	471692	PREDICTED: ribonuclease MC-like [*Cucumis sativus*]
CL30433Contig1	313638	Pectinesterase precursor, putative [*Ricinus communis*]
CL13820Contig1	240111	DS synthase [*Panax notoginseng*]
CL9626Contig2	217529	PREDICTED: catalase isozyme 1 isoform 1 [*Vitis vinifera*]
CL23464Contig1	213586	Hypothetical protein MTR_5g051130 [*Medicago truncatula*]
CL9511Contig1	196268	Cytochrome P450 CYP716A47 [*Panax ginseng*]
CL15802Contig1	164172	Thaumatin-like protein [*Actinidia deliciosa*]
