# Supplementary material for: Transcriptome analysis of leaves, roots and flowers of Panax notoginseng identifies genes involved in ginsenoside and alkaloid biosynthesis
Source: BMC Genomics. 2015 Apr 3;16(1):265. doi: 10.1186/s12864-015-1477-5 (PMC4399409; doi:10.1186/s12864-015-1477-5)
Supplement: Additional file 4: — Number of unigenes assigned to KEGG biochemical pathways in P. notoginseng. PDF document of the number of unigenes assigned to KEGG biochemical pathways. [file 12864_2015_1477_MOESM4_ESM.pdf]

Additional file 4 - Number of unigenes assigned to KEGG biochemical pathways in *P. notoginseng*

KEGG categories represented	Leaves	Roots	Flowers
**Metabolism**			
Carbohydrate metabolism	1,993	1,597	2,337
Energy metabolism	661	551	658
Lipid metabolism	882	724	841
Nucleotide metabolism	1,080	785	1,085
Amino acid metabolism	1,313	927	1,303
Metabolism of other amino acids	304	237	396
Glycan biosynthesis and metabolism	415	389	566
Metabolism of cofactors and vitamins	1,004	701	1,034
Biosynthesis of other secondary metabolites	350	278	412
Xenobiotics biodegradation and metabolism	360	281	479
**Genetic Information Processing**			
Translation	187	141	227
**Environmental Information Processing**			
Signal transduction	153	123	138
**Organismal Systems**			
Immune system	146	102	146
