# Supplementary material for: Transcriptome analysis of leaves, roots and flowers of Panax notoginseng identifies genes involved in ginsenoside and alkaloid biosynthesis
Source: BMC Genomics. 2015 Apr 3;16(1):265. doi: 10.1186/s12864-015-1477-5 (PMC4399409; doi:10.1186/s12864-015-1477-5)
Supplement: Additional file 8: — List of real-time PCR primer sequences. PDF document of the list of real-time PCR primer sequences. [file 12864_2015_1477_MOESM8_ESM.pdf]

Additional file 8 – List of real-time PCR primer sequences

Gene name	Primer (5'−3')	
	Forward	Reverse
Acetyl-CoA acetyltransferase	TCTAGGAAAGTTCGATGCTGCT	GCCACCAGTCTCCTTGAAAC
Hydroxymethyl glutaryl CoA synthase	GCTGGCACCATTTTCAACTT	CTTTTCAAGGTCCCGGCTA
Mevalonate kinase	AAACTTGCTACCAAATTGACAGG	TGGGTAACAGTGTCAGAACACA
Mevalonate diphosphosphate decarboxylase	CTTGGCACATGCAAAAGGTA	TTCAGTCCCTGACAATCTTCC
Isopentenylpyrophosphate isomerase	CTTCTCAAAAATTCTATCTCCCTCA	AAGAGATAGGCACCGGAGTTT
Farnesyl diphosphate synthase	TGCACTTGGTTGGTGCAT	CATAATATCATCAAGCACCAGAAAATA
Squalene synthase	ATGTCGATAAACTTGAGGACTTAAAAT	TCATTTAGGCACCGCACTG
Squalene epoxidase	TCTAGGAAAGTTCGATGCTGCT	GCCACCAGTCTCCTTGAAAC
Aspartate transaminase	TTCTGGCTTTTCCCTTCAAGT	CCGGCAGTAGATCCACATTT
Strictosidine synthase	ATCCGACGGGGTACAAAGTT	GATCATCGAGATCAAGCAAGC
Histidinol-phosphate transaminase	TGTCTCTGTCACCGGAAAAGT	CGTGATCATCTAGGGTTTGGA
CYP716A53v2	AAGCACCTCTCAATCCCAAG	GACACCATCACCAGAATCCA
Actin	TCGGACAACGAGGCAGCACTTT	GCTAAAGAGCAGCCAACAGGCC
